# Supplementary material for: Cryo-EM structures reveal native GABAA receptor assemblies and pharmacology
Source: Nature. 2023 Sep 20;622(7981):195–201. doi: 10.1038/s41586-023-06556-w (PMC10550821; doi:10.1038/s41586-023-06556-w)
Supplement: Supplementary file 1 — Supplementary Discussion, Methods and Figs. 1–7. [file 41586_2023_6556_MOESM1_ESM.pdf]

nature portfolio

https://doi.org/10.1038/s41586-023-06556-w

Supplementary information

Cryo-EM structures reveal native $GABA_A$ receptor assemblies and pharmacology

In the format provided by the authors and unedited

Supplementary Information

Cryo-EM structures reveal native $GABA_A$ receptor assemblies and pharmacology

Chang Sun[1], Hongtao Zhu[1,2], Sarah Clark[1], and Eric Gouaux[1,3]

[1]Vollum Institute, Oregon Health and Science University, 3232 SW Research Drive, Portland, Oregon 97239.

[2]Present address: Institute of Physics, Chinese Academy of Sciences, Beijing, China

[3]Howard Hughes Medical Institute, Oregon Health and Science University, 3232 SW Research Drive, Portland, Oregon 97239.

[3]Correspondence to Eric Gouaux: gouauxe@ohsu.edu

Table of Contents

SUPPLEMENTARY DISCUSSION **3**

GABA$_A$R subunit assignment based on cryo-EM density maps 3

SUPPLEMENTARY METHODS **5**

Isotope-dilution quantification of allopregnanolone using LC-MS/MS 5

SUPPLEMENTARY FIGURES **7**

Supplementary Figure 1 | Distinctive *N*-glycosylation among GABA$_A$R subunits 7

Supplementary Figure 2 | Inter-domain rearrangements 8

Supplementary Figure 3 | PDB 6I53 represents a GABA bound conformation 9

Supplementary Figure 4 | Lipid-like densities 10

Supplementary Figure 5 | Mass-spec detection of the endogenous neurosteroid 11

Supplementary Figure 6 | Pore profile comparisons 13

Supplementary Figure 7 | GABA binding with partial loop C closure 14

SUPPLEMENTARY REFERENCES **15**

Supplementary Discussion

$GABA_AR$ subunit assignment based on cryo-EM density maps

$GABA_AR$ subunits are classified into subgroups (α, β, γ, ϱ, δ, ε, π, θ) and based on the established tissue distribution and on our mass spec data, we have restricted our assignment to subunits belonging to the α, β, γ, δ subgroups. These subgroups have distinctive, characteristic glycosylation patterns (**Supplementary Figure 1**). For instance, only α subunits contain glycosylation within the extracellular domain vestibule while β subunits show two notable glycosylation chains at the extracellular domain periphery. These glycosylation structural features can be readily recognized at resolution around 8 Å and can be used to distinguish different $GABA_AR$ subgroups at intermediate resolution.

Within each subgroup, however, distinguishing subunits is more challenging and requires visible difference at the amino acid residue level, typically requiring a resolution of the cryo-EM map at 3 Å or higher. To be certain with assignment, we have examined the density map extensively but limited our assessment to positions where the residue placements are without ambiguity and where the residue sequences are distinctive between subunits (more than 3 carbon atoms or one sulfur atom between different subunits). The resulting evidence is summarized in the **Extended Data Figure 6**, and we provide our work-through of these examples below, with each numbered item below corresponding to one row of cryo-EM density map comparison from **Extended Data Figure 6**.

1. The side chain density at position R63 (α1 numbering before the signal peptide cleavage) strongly supports the subunit identity of chains A and C of the two-Fab as α1 or α5 (α1/α5), chain A of the meta-one-Fab as α1/α5, chain C of the meta-one-Fab as α2/α3/α4/α6, chain A of the ortho-one-Fab as α2/α3/α4/α6, and chain C of the ortho-one-Fab as α1/α5.
2. The side chain density at position M140 supports the subunit identity of chain A and C of the two-Fab as α1/α2, chain A of the meta-one-Fab as α1/α2, chain C of the meta-one-Fab as α3/α4/α5/α6, chain A of the ortho-one-Fab as α3/α4/α5/α6, and chain C of the ortho-one-Fab as α1/α2.
3. The side chain density at position L145 strongly supports the subunit identity of all three receptors as α1/α2/α3/α5, but not α4/α6 for chain A and chain C. Combining these

pieces of side chain information and Fab binding, we assign the chain A of the two-Fab as α1 and the chain C of the two-Fab as α1, the chain A of the meta-one-Fab as α1 and the chain C of the meta-one-Fab as α2/α3 (α3 has a better overall fit), the chain A of the ortho-one-Fab as α2/α3 (α3 has a better overall fit) and the chain C of the meta-one-Fab as α1.

4. The side chain density at position A90 (β2 numbering before the signal peptide cleavage) strongly supports the subunit identity of chain B and chain E of all three receptors as β1/β2, but not β3.
5. The side chain density at position R193 supports the subunit identity of chain B and chain E of the two-Fab as not β1, and chain B and chain E of the meta-one-Fab and ortho-one-Fab as containing β1. To conclude, we assign the chain B of the two-Fab as β2 and the chain E of the two-Fab as β2, the chain B of the meta-one-Fab as β1/β2 and the chain E of the meta-one-Fab as β1/β2, the chain B of the ortho-one-Fab as β1/β2 and the chain E of the ortho-one-Fab as β1/β2.
6. The side chain density at positions H92 and M95 (γ2 numbering before the signal peptide cleavage) supports the subunit identity of chain D of all three receptors as γ2.
7. The side chain density at position F115 strongly supports the subunit identity of chain D of all three receptors as not γ1 and, therefore, likely to be γ2 or γ3.
8. The side chain density at position Y121 strongly supports the subunit identity of chain D of all three receptors as not γ3 and, therefore, likely to be γ1 or γ2. To conclude, we assign the chain D of the two-Fab as γ2, the chain D of the meta-one-Fab as γ2, and the chain D of the ortho-one-Fab as γ2.
9. Consistent with our subunit assignment is the fucose density at the α2/3 subunits of the one-Fab receptors but not at the α1 subunits of the one-Fab receptors or the two-Fab receptor.

Supplementary Methods

Isotope-dilution quantification of allopregnanolone using LC-MS/MS

Neurosteroids (allopregnanolone, epipregnanolone, isopregnanolone, pregnanolone) and isotope-labeled internal standard allopregnanolone-d5 were purchased from Toronto Research Chemicals (Toronto, ON, Canada). The O-(3-trimethylammonium-propyl) hydroxylamine quaternary amonoxy (QAO) reagent used for derivatization was in the form of Amplifex Keto reagent kit from AB Sciex (Framingham, MA). Solvents for liquid chromatography-tandem mass spectrometry (LC-MS/MS) analysis were from VWR (Tualatin, OR).

Neurosteroid stocks and internal standards (INST) were prepared in methanol. Stocks (5 μL) and the INST allopregnanolone-d5 (5 μL) were mixed with PBS (95 μL) to prepare standard samples with final concentrations ranging from 0.05 to 100 ng/ml. All standards and samples were treated with 1000 μl of acetonitrile, vortexed and mixed using Benchmark Multi-Thermo heat/shaker at 1500 rpm at 22ºC for 5 mins, and centrifuged to remove protein at 12,000 g for 5 mins. The supernatant was dried under vacuum and then treated with 75 μl of derivatization reagent. The keto moiety was derivatized with QAO reagent to form a cationic oxime derivative to enable highly sensitive LC-ESI-MS/MS quantification of neurosteroids. The working derivatization reagent was prepared according to vendor instructions. The derivatized samples were diluted 1:4 with 5% acetic acid in methanol before LC-MS/MS analysis. The supernatant was placed in sample vials for analysis by LC-MS/MS using an injection volume of 5 μl. The lower limit of quantification of allopregnanolone was 75 pg/ml, with an accuracy of 101% and a precision of 2.2%.

The samples with INST were analyzed using a Sciex 4000 QTRAP hybrid/triple quadrupole linear ion trap mass spectrometer (Foster City, CA) with electrospray ionization (ESI) in the positive mode. The mass spectrometer was interfaced to a Shimadzu HPLC system (Columbia, MD) with SIL-20AC XR auto-sampler, LC-20AD XR LC pumps, and CTO-20AC column oven. Compounds were quantified with multiple reaction monitoring (MRM) and the MS/MS transitions used were optimized for sensitivity by infusion of pure derivatized compounds with method settings. The transition used for quantification of QAO-allopregnanolone was m/z 433.3>126.1 with m/z 433.3>374.3 used for peak qualification to ensure method specificity. The transition used for quantification of QAO-allopregnanolone-d5

was m/z 438.3>126. Allopregnanolone was separated from interferents using a Luna 5u C8(2) 50x2 mm column (Phenomenex) kept at 35 °C using a column oven. The gradient mobile phase was delivered at a flow rate of 0.8 ml/min and consisted of two solvents: solvent A (0.1% formic acid in water) and solvent B (0.1% formic acid in acetonitrile). The initial concentration of solvent B was 20%, followed by a linear increase to 60% B in 10 min, then to 95% B in 0.1 min, held for 3 minutes, decreased back to starting 20% B over 0.1 min, and then held for 2 min. The retention time was 3.99 min for allopregnanolone and pregnanolone, 3.64 min for isopregnanolone, and 3.61 min for epipregnanolone. Data were acquired using Analyst 1.6.2 and analyzed with MultiQuant 3.0.3 software.

To further distinguish allopregnanolone and pregnanolone, a different HPLC condition was used. In this case, a Poroshell 120 EC-C18 100x2.1 mm 2.7um column (Agilent) was kept at 35 °C using a column oven. The gradient mobile phase was delivered at a flow rate of 0.4 ml/min (0–5.9min), 0.2ml/min (6.0–8.9min), and 0.4ml/min (9–15min), and consisted of two solvents, A: 0.1% formic acid in water, B: 0.1% formic acid in acetonitrile. The initial concentration of solvent B was 30%, followed by a linear increase to 52% B in 6.5 min, held for 2.5min, then to 95% B in 0.1 min, held for 2.9 minutes, decreased back to starting 30% B over 0.1 min, and then held for 2.9 min. The retention time for allopregnanolone was 6.4 min, pregnenolone was 6.2 min, and 3α-allopregnanolone-d5 was 6.3 min.

Supplementary Figures

Supplementary Figure 1 | Distinctive *N*-glycosylation among GABA$_A$R subunits

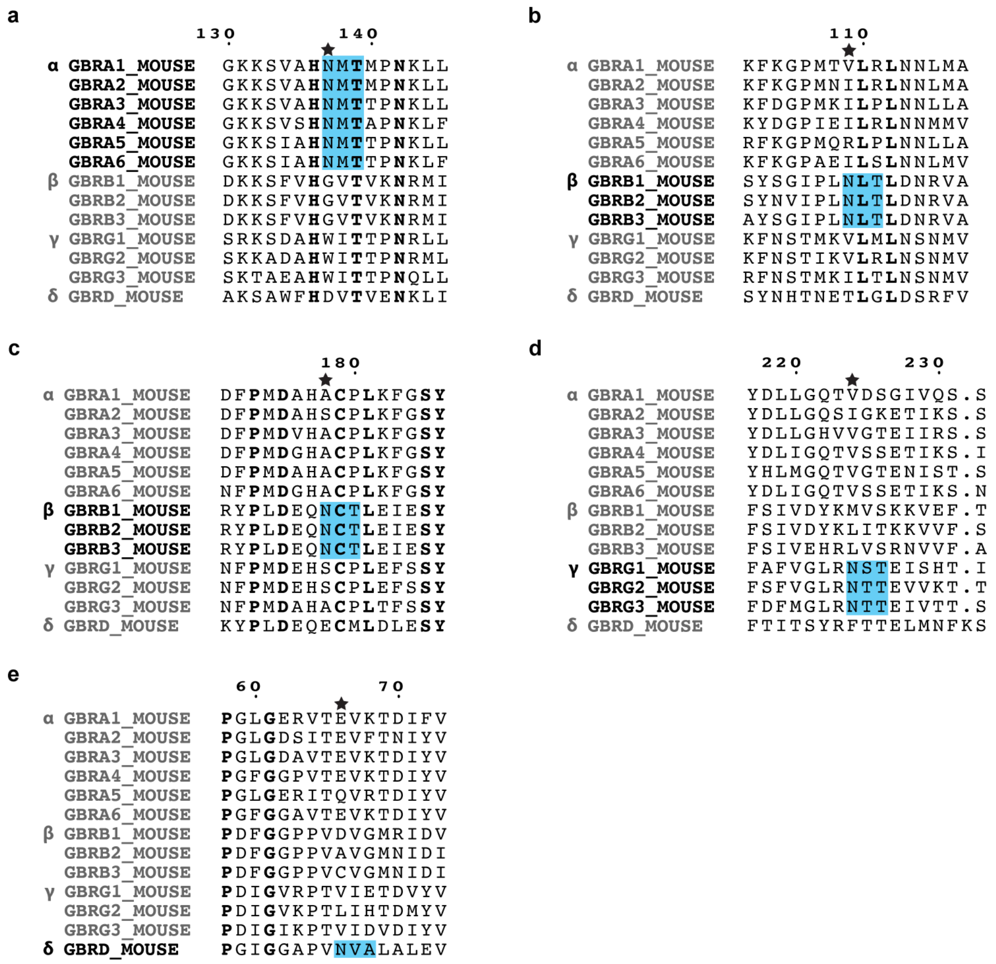

Supplementary Figure 1. *N*-glycosylation unique to a subgroup of GABA$_A$R subunits. Sequences of the murine α/β/γ/δ subunits are aligned, and sequence alignments surrounding the N-glycosylation that has been verified with structure studies[1-4] are shown (a sequence window of 16-amino acid residue), with the α subunit position shown in **a**, β subunit positions shown in **b** and **c**, γ subunit position shown in **d**, and δ subunit position shown **e**. Sequence alignment was performed with Clustal Omega[5] and rendered with ESPript[6]. Strictly conserved residues across all subunits are shown in bold. Consensus *N*-glycosylation motifs (Asn-X-Ser/Thr, X denotes an amino acid residue other than proline) are boxed in blue, while the positions of the glycosylated Asn are marked with a star symbol on the top of the sequence alignments. Residue numberings shown are based on the murine α1 subunit before the signal peptide cleavage.

Supplementary Figure 2 | Inter-domain rearrangements

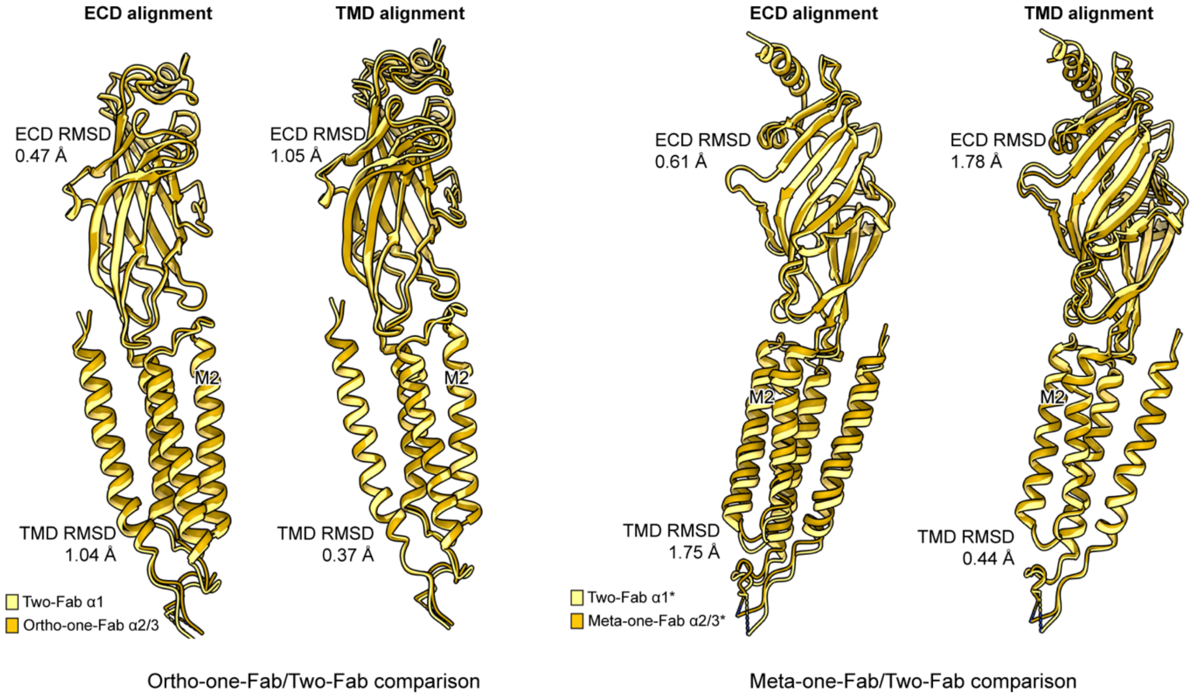

Supplementary Figure 2. Inter-domain rearrangements between two-Fab $GABA_A$Rs and the one-Fab $GABA_A$Rs (ortho-one-Fab and meta-one-Fab) from the APG/GABA dataset. The two-Fab $\alpha1^*$-$\beta2$-$\alpha1$-$\beta2^*$-$\gamma2$ (* denotes the subunit is next to a γ subunit, subunits are counted clockwise when viewed from the extracellular space) is compared with the ortho-one-Fab $\alpha1^*$-$\beta1/2$-$\alpha2/3$-$\beta1/2^*$-$\gamma2$ on the left, and with the meta-one-Fab $\alpha2/3^*$-$\beta1/2$-$\alpha1$-$\beta1/2^*$-$\gamma2$ on the right. The α1 and α2/3 subunits from the equivalent positions are structurally superimposed (in ChimeraX[7]), either with the extracellular domain (ECD) or the transmembrane domain (TMD). The root mean square deviation (RMSD) of the amino acid backbones are reported for individual domains. For the ECD, the residue ranges are 12 to 221 for α1, 37 to 246 for α2/3 (with residue numbers specific to the α3 subunit). For the TMD, the residue ranges are 222–246, 250–275, 284–309, 390–415 for α1, and 247–271, 275–300, 309–334, 426–451 for α2/3 (with residue numbers specific to the α3 subunit). The higher RMSD of ECD when the structures are aligned with TMD, and vice versa, demonstrates the domain rearrangements between the two-Fab receptors and the one-Fab receptors.

Supplementary Figure 3 | PDB 6I53 represents a GABA bound conformation

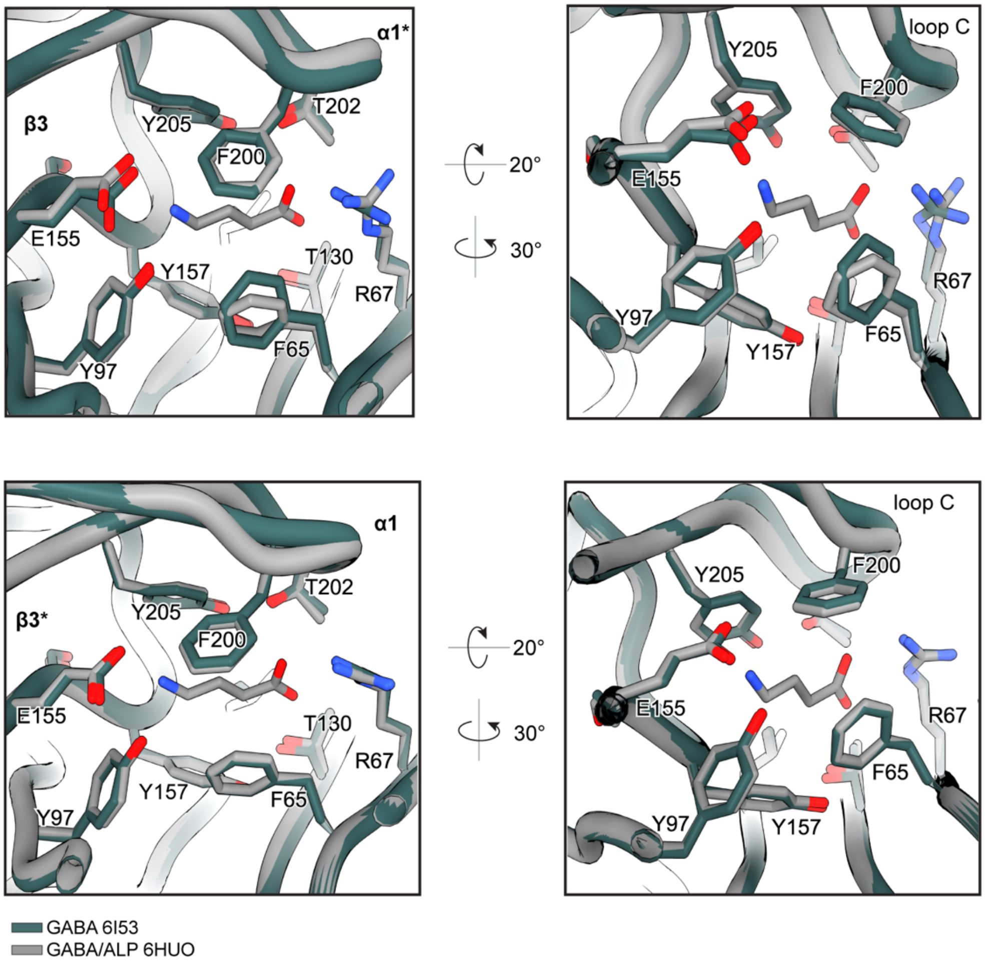

Supplementary Figure 3. Structural comparison between 6I53 and 6HUO. The structures (α1β3γ2 6I53[3] shown in dark gray, α1β3γ2 6HUO[8] shown in gray) are superimposed based on the GABA binding pockets. Specific residues include F65, R67, L118, T130 from the α1 subunit and Y97, E155, S156, Y157, F200, T202, Y205 from the β3 subunit. These residues along with the GABA molecules from 6HUO are shown in stick representation. Of note, both the 6I53 and the 6HUO structures exhibit a complete loop C closure. Furthermore, the GABA binding pockets in 6I53 closely resemble those in 6HUO. The $\beta3^{+}/\alpha1^{*-}$ pocket has a backbone RMSD of 0.23 Å and an all-atom RMSD of 0.37 Å while the $\beta3^{*+}/\alpha^{-}$ pocket has a backbone RMSD of 0.29 Å and an all-atom RMSD of 0.25 Å. These findings suggest that the 6I53 structure represents a GABA-bound conformation, despite being initially modeled without GABA.

Supplementary Figure 4 | Lipid-like densities

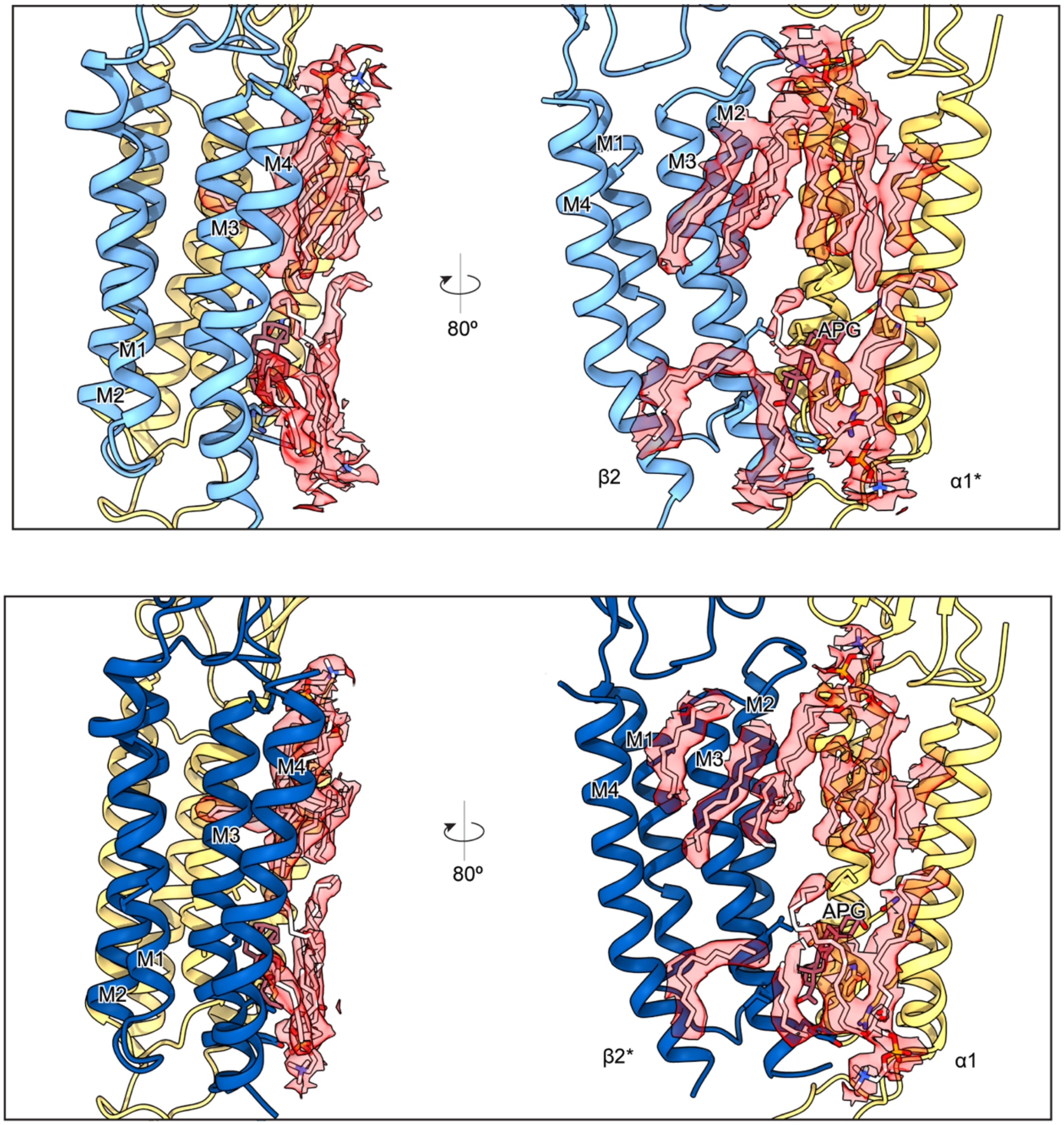

Supplementary Figure 4. Lipid-like densities resolved near the allopregnanolone binding pockets. The lipid-like densities were modeled as nine lipid molecules at the β2/α1* interface and ten lipid molecules at the β2*/α1 interface. These modeled molecules include octane, dodecane, and palmitoyl-oleoyl-phosphocholine (POPC). These assignments are based on the density shapes and modeling convenience and may not be an accurate representation of their molecular identities. This figure shows the cryo-EM map at a threshold of 3.5 σ. Note one of the acyl chains of the modeled POPC at the upper leaflet is inserted deeply into the anesthetic binding pocket[3,9] between the TMDs of β2 and α1 subunits.

Supplementary Figure 5 | Mass-spec detection of the endogenous neurosteroid

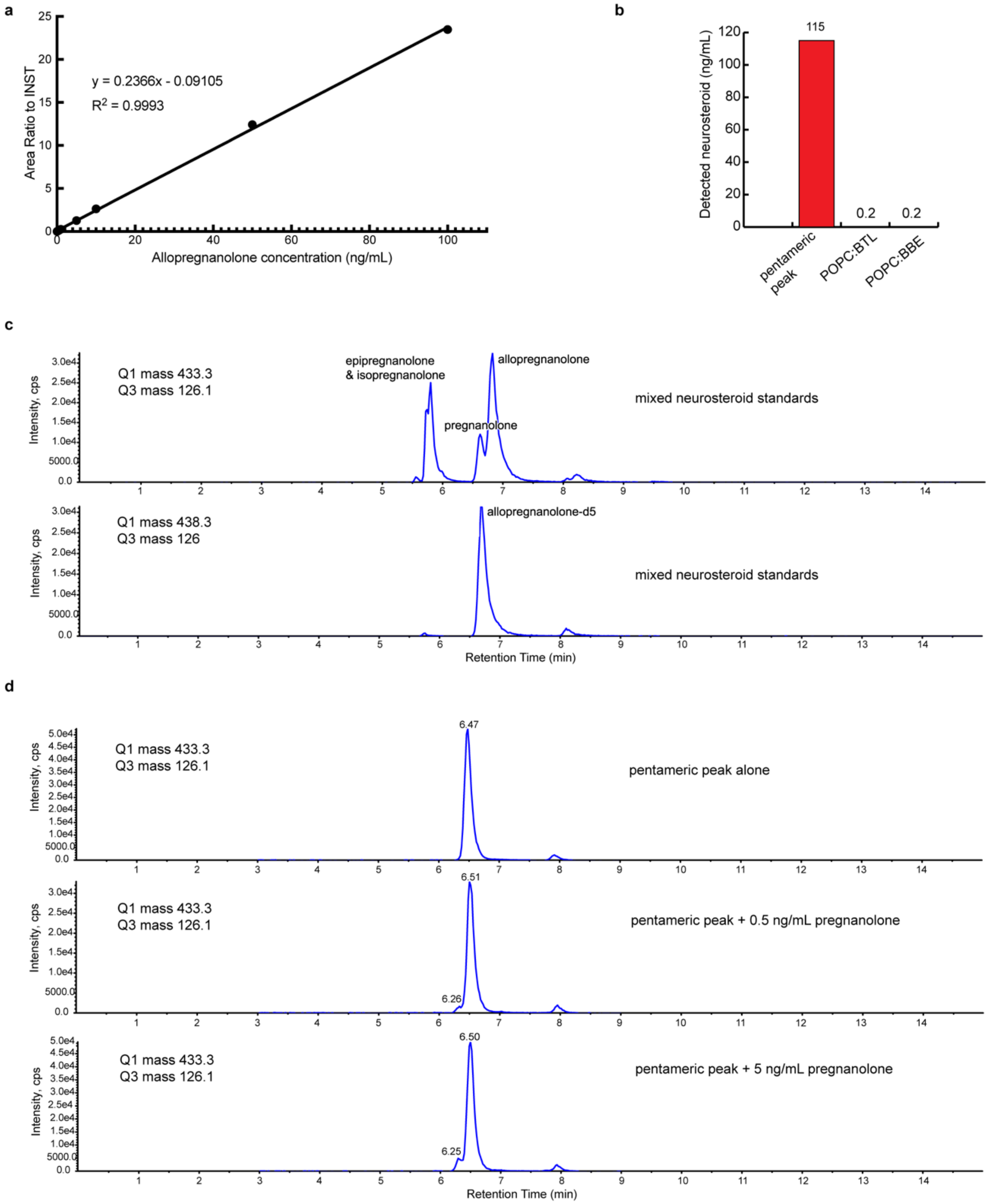

Supplementary Figure 5. Mass spectrometry analysis of neurosteroid for the ZOL/GABA sample. **a**, Standard curve of allopregnanolone quantitation based on isotope dilution. **b**, Quantitation of neurosteroid in the pentameric sample and lipid stocks (85:15 mixture of POPC with either brain total

lipids from Avanti or bovine brain extracts from Sigma) used for on-column nanodisc reconstitution. Considering the volume, the neurosteroid from the exogenous lipid makes up only 0.3% of the detected neurosteroid in the protein sample. **c**, Chromatographs of the mixed neurosteroid standards under the final LC condition. **d**, Chromatographs of protein sample alone or spiked with different amounts of pregnanolone standard.

Supplementary Figure 6 | Pore profile comparisons

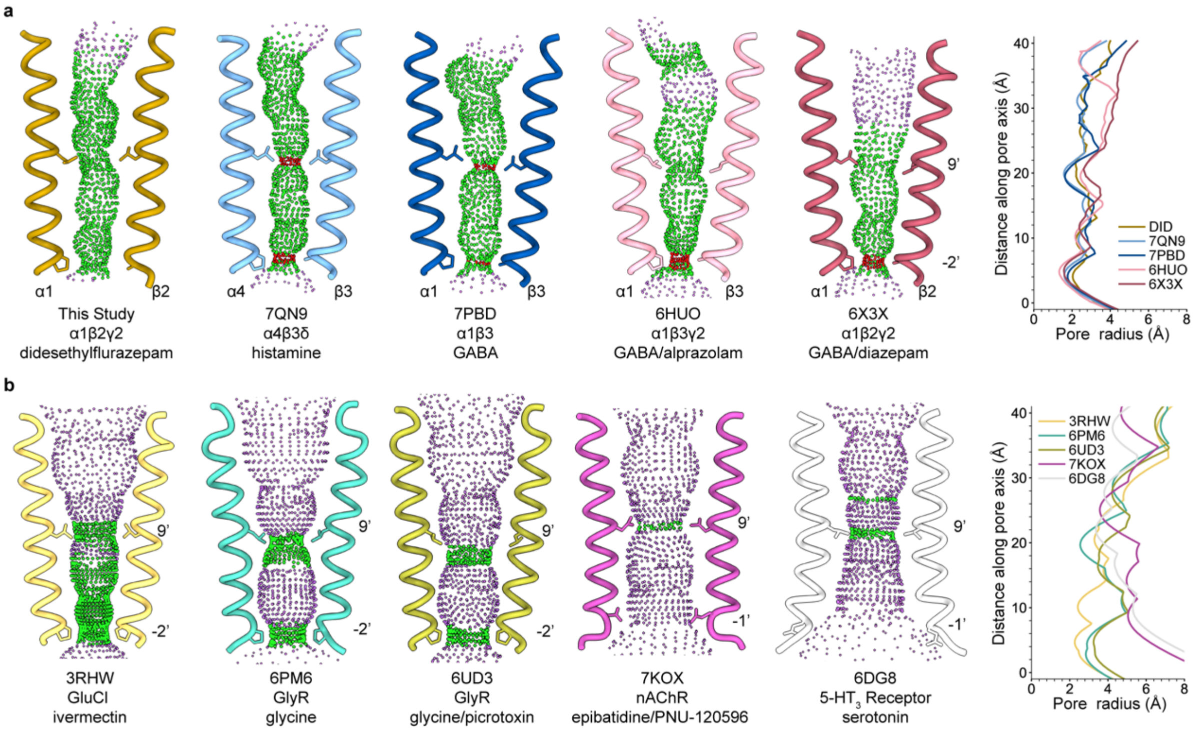

Supplementary Figure 6. Channel pore comparison. a, Shown are pore profiles of selected $GABA_A R$ structures and the two-Fab didesethylflurazepam (DID) structure. From left to right are pore profiles of the two-Fab DID from this study, α4-β3-β3-β3-δ receptor (subunits are counted clockwise when viewed from the extracellular space) with histamine bound (7QN9)[4], α1-β3-α1-β3-β3 receptor with GABA bound (7PBD)[10], α1-β3-α1-β3-γ2 receptor with GABA and alprazolam bound (6HUO)[8], and α1-β2-α1-β2-γ2 receptor with GABA and diazepam bound (6X3X)[9]. **b,** Pore comparison between open structures from the Cys-loop family. From left to right are pore profiles of glutamate-gated chloride channel bound with ivermectin (3RHW)[11], glycine receptor bound with glycine (6PM6)[12], glycine receptor bound with glycine and picrotoxin (6UD3)[13], nicotinic acetylcholine receptor bound with epibatidine and PNU-120596 (7KOX)[14], and $5\text{-}HT_3$ receptor bound with serotonin (6DG8)[15], all of which are homopentamers. Pore profiles were calculated using the HOLE[16] program; blue, green, and red spheres define radii of >4 Å, 1.8–4 Å, and <1.8 Å, respectively. Residues at the two constriction gates (9' and -2' with the exception of nAChR and $5\text{-}HT_3$ Receptor, which have gates at 9' and -1' positions) of the channel are shown, which roughly correspond to the 20 Å and the 5 Å positions in the consolidated plots on the right.

Supplementary Figure 7 | GABA binding with partial loop C closure

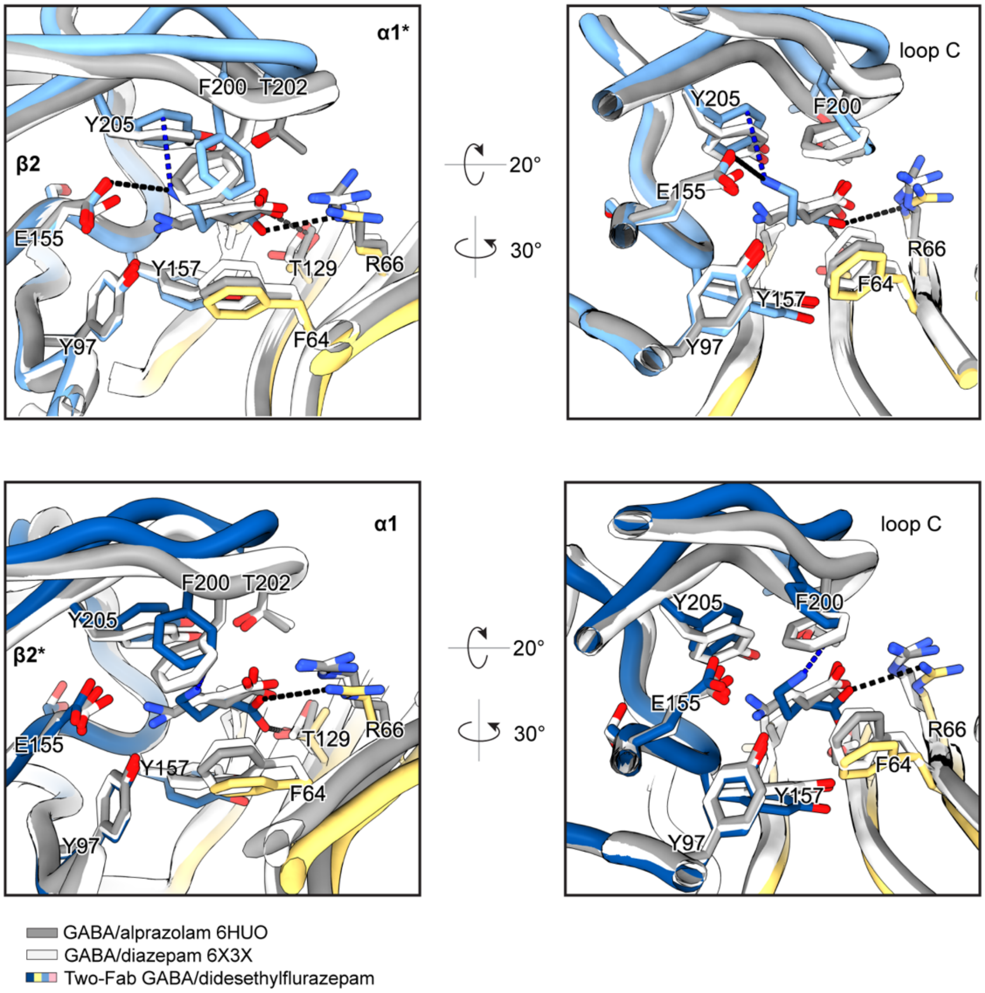

Supplementary Figure 7. Structural comparison between previous GABA/benzodiazepine structures and the two-Fab GABA/didesethylflurazepam structure. The structures (α1β3γ2 6HUO[8] shown in gray, α1β2γ2 6X3X[9] shown in white, two-Fab DID shown in colors) are superimposed based on the GABA binding pockets, including F64, R66, L117, T129 from the α1 subunit and Y97, E155, S156, Y157, F200, T202, Y205 from the β2/β3 subunit (residue numberings are based on the native receptor from this work). Hydrogen bonds are shown as black dashed lines while π-cation interactions are shown as blue dashed lines. Compared to previous structures with saturating amounts of GABA, the two-Fab DID structure solved with the endogenous GABA only shows an incomplete loop C closure, a less compact GABA binding pocket, and fewer hydrogen bonds and π-cation interactions between the GABA ligand and the protein matrix. In the meantime, the GABA molecule adopts a less extended conformation in the DID structure.

Supplementary References

1 Phulera, S., Zhu, H., Yu, J., Claxton, D. P., Yoder, N., Yoshioka, C. & Gouaux, E. Cryo-EM structure of the benzodiazepine-sensitive α1β1γ2S tri-heteromeric $GABA_A$ receptor in complex with GABA. *eLife* **7**, e39383, (2018).

2 Zhu, S., Noviello, C. M., Teng, J., Walsh, R. M., Jr., Kim, J. J. & Hibbs, R. E. Structure of a human synaptic $GABA_A$ receptor. *Nature* **559**, 67–72, (2018).

3 Laverty, D., Desai, R., Uchański, T., Masiulis, S., Stec, W. J., Malinauskas, T., Zivanov, J., Pardon, E., Steyaert, J., Miller, K. W. & Aricescu, A. R. Cryo-EM structure of the human α1β3γ2 $GABA_A$ receptor in a lipid bilayer. *Nature* **565**, 516–520, (2019).

4 Sente, A., Desai, R., Naydenova, K., Malinauskas, T., Jounaidi, Y., Miehling, J., Zhou, X., Masiulis, S., Hardwick, S. W., Chirgadze, D. Y., Miller, K. W. & Aricescu, A. R. Differential assembly diversifies $GABA_A$ receptor structures and signalling. *Nature* **604**, 190–194, (2022).

5 Sievers, F., Wilm, A., Dineen, D., Gibson, T. J., Karplus, K., Li, W., Lopez, R., McWilliam, H., Remmert, M., Söding, J., Thompson, J. D. & Higgins, D. G. Fast, scalable generation of high-quality protein multiple sequence alignments using Clustal Omega. *Mol. Syst. Biol.* **7**, 539, (2011).

6 Robert, X. & Gouet, P. Deciphering key features in protein structures with the new ENDscript server. *Nucleic Acids Res.* **42**, W320-324, (2014).

7 Pettersen, E. F., Goddard, T. D., Huang, C. C., Meng, E. C., Couch, G. S., Croll, T. I., Morris, J. H. & Ferrin, T. E. UCSF ChimeraX: Structure visualization for researchers, educators, and developers. *Protein Sci.* **30**, 70–82, (2021).

8 Masiulis, S., Desai, R., Uchański, T., Serna Martin, I., Laverty, D., Karia, D., Malinauskas, T., Zivanov, J., Pardon, E., Kotecha, A., Steyaert, J., Miller, K. W. & Aricescu, A. R. $GABA_A$ receptor signalling mechanisms revealed by structural pharmacology. *Nature* **565**, 454–459, (2019).

9 Kim, J. J., Gharpure, A., Teng, J., Zhuang, Y., Howard, R. J., Zhu, S., Noviello, C. M., Walsh, R. M., Lindahl, E. & Hibbs, R. E. Shared structural mechanisms of general anaesthetics and benzodiazepines. *Nature* **585**, 303–308, (2020).

10 Kasaragod, V. B., Mortensen, M., Hardwick, S. W., Wahid, A. A., Dorovykh, V., Chirgadze, D. Y., Smart, T. G. & Miller, P. S. Mechanisms of inhibition and activation of extrasynaptic αβ $GABA_A$ receptors. *Nature* **602**, 529–533, (2022).

11 Hibbs, R. E. & Gouaux, E. Principles of activation and permeation in an anion-selective Cys-loop receptor. *Nature* **474**, 54–60, (2011).

12 Yu, J., Zhu, H., Lape, R., Greiner, T., Du, J., Lü, W., Sivilotti, L. & Gouaux, E. Mechanism of gating and partial agonist action in the glycine receptor. *Cell* **184**, 957–968, (2021).

13 Kumar, A., Basak, S., Rao, S., Gicheru, Y., Mayer, M. L., Sansom, M. S. P. & Chakrapani, S. Mechanisms of activation and desensitization of full-length glycine receptor in lipid nanodiscs. *Nat Commun* **11**, 3752, (2020).

14 Noviello, C. M., Gharpure, A., Mukhtasimova, N., Cabuco, R., Baxter, L., Borek, D., Sine, S. M. & Hibbs, R. E. Structure and gating mechanism of the α7 nicotinic acetylcholine receptor. *Cell* **184**, 2121–2134, (2021).

15 Basak, S., Gicheru, Y., Rao, S., Sansom, M. S. P. & Chakrapani, S. Cryo-EM reveals two distinct serotonin-bound conformations of full-length 5-HT(3A) receptor. *Nature* **563**, 270–274, (2018).

16 Smart, O. S., Neduvelil, J. G., Wang, X., Wallace, B. A. & Sansom, M. S. HOLE: a program for the analysis of the pore dimensions of ion channel structural models. *J. Mol. Graph.* **14**, 354–360, (1996).
