# Supplementary material for: Cryo-EM structures reveal native GABAA receptor assemblies and pharmacology
Source: Nature. 2023 Sep 20;622(7981):195–201. doi: 10.1038/s41586-023-06556-w (PMC10550821; doi:10.1038/s41586-023-06556-w)
Supplement: Supplementary file 2 — Reporting Summary [file 41586_2023_6556_MOESM2_ESM.pdf]

nature portfolio

Corresponding author(s): Eric Gouaux

Last updated by author(s): Jul 28, 2023

Reporting Summary

Nature Portfolio wishes to improve the reproducibility of the work that we publish. This form provides structure for consistency and transparency in reporting. For further information on Nature Portfolio policies, see our Editorial Policies and the Editorial Policy Checklist.

Statistics

For all statistical analyses, confirm that the following items are present in the figure legend, table legend, main text, or Methods section.

n/a	Confirmed	
☐	☒	The exact sample size (*n*) for each experimental group/condition, given as a discrete number and unit of measurement
☐	☒	A statement on whether measurements were taken from distinct samples or whether the same sample was measured repeatedly
☒	☐	The statistical test(s) used AND whether they are one- or two-sided<br>*Only common tests should be described solely by name; describe more complex techniques in the Methods section.*
☒	☐	A description of all covariates tested
☒	☐	A description of any assumptions or corrections, such as tests of normality and adjustment for multiple comparisons
☐	☒	A full description of the statistical parameters including central tendency (e.g. means) or other basic estimates (e.g. regression coefficient) AND variation (e.g. standard deviation) or associated estimates of uncertainty (e.g. confidence intervals)
☒	☐	For null hypothesis testing, the test statistic (e.g. *F*, *t*, *r*) with confidence intervals, effect sizes, degrees of freedom and *P* value noted<br>*Give P values as exact values whenever suitable.*
☒	☐	For Bayesian analysis, information on the choice of priors and Markov chain Monte Carlo settings
☒	☐	For hierarchical and complex designs, identification of the appropriate level for tests and full reporting of outcomes
☒	☐	Estimates of effect sizes (e.g. Cohen's *d*, Pearson's *r*), indicating how they were calculated

Our web collection on statistics for biologists contains articles on many of the points above.

Software and code

Policy information about availability of computer code

Data collection	SerialEM 3.8, MicroBeta workstation v 4.0, Leica Application Suite X v3.7.4, Labsolutions v5.11,
Data analysis	RELION-3.1, cryosparc v3.3.1, Prism-9 v9.5.1, Coot v0.9-pre, Phenix v1.20.1, Chimera v1.16, ChimeraX v1.4, Python v3.8.13, locScale v0.1 (Github commit fe5d7e7), deepEMhancer (Github commit 2817c29), TIRF photobleaching analysis script (https://doi.org/10.5281/zenodo.8161179)

For manuscripts utilizing custom algorithms or software that are central to the research but not yet described in published literature, software must be made available to editors and reviewers. We strongly encourage code deposition in a community repository (e.g. GitHub). See the Nature Portfolio guidelines for submitting code & software for further information.

Data

Policy information about availability of data

All manuscripts must include a data availability statement. This statement should provide the following information, where applicable:

- Accession codes, unique identifiers, or web links for publicly available datasets
- A description of any restrictions on data availability
- For clinical datasets or third party data, please ensure that the statement adheres to our policy

The cryo-EM maps and coordinates for the native GABA receptor in complex with didesethylflurazepam and endogenous GABA (two-Fab-DID) have been deposited in the Electron Microscopy Data Bank (EMDB) under accession number EMD-29728 and in the Protein Data Bank (PDB) under accession code 8G4O. The cryo-EM

maps and coordinates for the native GABA receptor in complex with zolpidem, GABA, and endogenous neurosteroids have been deposited and accessed via EMD-39727/8G4N (two-Fab-ZOL), EMD-29743/8G5H (ortho-one-Fab-ZOL), EMD-29742/8G5G (meta-one-Fab-ZOL). The cryo-EM maps and coordinates for the native GABA receptor in complex with GABA, and allopregnanolone have been deposited and accessed via EMD-29350/8FOI (two-Fab-ALP), EMD-29741/8G5F (ortho-one-Fab-ALP), EMD-29733/8G4X (meta-one-Fab-ALP).

Human research participants

Policy information about studies involving human research participants and Sex and Gender in Research.

Reporting on sex and gender	N/A
Population characteristics	N/A
Recruitment	N/A
Ethics oversight	N/A

Note that full information on the approval of the study protocol must also be provided in the manuscript.

Field-specific reporting

Please select the one below that is the best fit for your research. If you are not sure, read the appropriate sections before making your selection.

☒ Life sciences ☐ Behavioural & social sciences ☐ Ecological, evolutionary & environmental sciences

For a reference copy of the document with all sections, see nature.com/documents/nr-reporting-summary-flat.pdf

Life sciences study design

All studies must disclose on these points even when the disclosure is negative.

Sample size	Sample sizes of cryo-EM data were determined by the anticipation of particle numbers within the constraints of microscope availability. No calculation of sample size was performed beforehand. We opted for a sample size of three, because it was done previously and we thought it is a good compromise between statistics and practicality. Single-molecule photobleaching experiments were carried out by collecting at least three photobleaching movies from different sample chambers. Three replicates of each condition were used for the radio-ligand binding assay.
Data exclusions	No data were excluded from the analyses.
Replication	Cryo-EM related biochemical experiments, including protein purification, SDS-PAGE analysis, and Western blot were repeated at least three times. Despite some degree of variability in protein yield, the pattern of proteins observed in SDS-PAGE and the detection of proteins with Western blot were reproducible. Radio ligand binding assay were repeated at least twice with independent samples on different days. This assay consistently yielded comparable binding affinities, reaffirming its reproducibility. The single-molecule photobleaching control experiment with isolated 8E3-Fab were repeated with two independent samples on different days with success. The single-molecule photobleaching experiments with 8E3-Fab bound GABAA receptors was conducted once. Because we obtained consistent results from the three parallel trials, which consisted of ~150 photobleaching traces each, no replication attempts have been made for this particular experiment. Finally, mass spectrometry analysis was conducted on independent samples using both the mass spectrometry core facilities at OHSU and LSU. Despite the difference in instrumentation, the identified proteins were largely consistent.
Randomization	For the single-molecule photobleaching experiments, photobleaching movies were acquired at random regions of the sample chamber. We did not employ randomization for sample or organism allocation across our range of other experiments, which include protein purification, gel analysis, ligand binding assay, cryo-EM data collection and analysis, as well as mass spectrometry analysis. This approach is primarily due to the fact that these specific types of experiments do not necessitate randomization or control for covariates, as they are not influenced by the sample allocation process common in other research methods. The only potential exception to this is our cryo-EM data collection where we selected squares based on subjective criteria of ice thickness and particle distribution, which can be argued as a form of non-random sample selection. However, it is important to note that this selection process is widely accepted in the field because it is a crucial step to ensure usable, high-quality data.
Blinding	The investigators were not blinded. In the context of our research, blinding during data collection/analysis was not feasible or relevant for a number of reasons. Specifically, for cryo-EM analysis, the goal is to describe the structure of the sample and the sample details should be given as a prior. As for the mass spectrometry analysis, single-molecule experiments, ligand binding assays, while it is technically feasible to conduct with the investigators blinded, the practicality is questionable. Introducing blinding would increase the labor cost substantially, without significantly enhance the validity of the results, as the downstream protein detection, determination of photobleaching steps, or curve fitting are reasonably streamlined.

Reporting for specific materials, systems and methods

We require information from authors about some types of materials, experimental systems and methods used in many studies. Here, indicate whether each material, system or method listed is relevant to your study. If you are not sure if a list item applies to your research, read the appropriate section before selecting a response.

Materials & experimental systems

n/a	Involved in the study
☐	☒ Antibodies
☐	☒ Eukaryotic cell lines
☒	☐ Palaeontology and archaeology
☐	☒ Animals and other organisms
☒	☐ Clinical data
☒	☐ Dual use research of concern

Methods

n/a	Involved in the study
☒	☐ ChIP-seq
☒	☐ Flow cytometry
☒	☐ MRI-based neuroimaging

Antibodies

Antibodies used	In house antibody: 8E3-GFP Fab anti-GBRA1 (produced by our lab), biotinylated anti-GFP nanobody (plasmid of the GFP nanobody was a gift from Brett Collins, and the nanobody was expressed, purified and biotinylated in our lab)<br>Commercial antibodies: anti-GBRA1 (Millipore, 06–868), anti-NL2 (Synaptic Systems, 129 202).
Validation	Validation of 8E3 mAb and its papain-digested fragment can be found in the previous paper from our lab (Phulera, S., Zhu, H., Yu, J., Claxton, D. P., Yoder, N., Yoshioka, C. & Gouaux, E. Cryo-EM structure of the benzodiazepine-sensitive α1β1γ2S tri-heteromeric GABAA receptor in complex with GABA. eLife 7, e39383, (2018))<br>Validation of the recombinant 8E3-GFP can be found in this study in the Extended Data Figure 1.<br>Validation of the anti-GFP nanobody can be found in the publication (Kubala, M. H., Kovtun, O., Alexandrov, K. & Collins, B. M. Structural and thermodynamic analysis of the GFP:GFP-nanobody complex. Protein Sci. 19, 2389-2401, (2010)). The biotinylation of the anti-GFP nanobody was carried out according to the manufacturer instructions (ThermoFisher A39259) and not validated.<br>The validation of commercial antibodies for western blot can be found in vendors' websites: https://www.emdmillipore.com/US/en/product/Anti-GABAA-Receptor-1-Antibody,MM_NF-06-868?ReferrerURL=https%3A%2F%2Fwww.google.com%2F (anti-GBRA1), https://sysy.com/product/129202 (anti-NL2).

Eukaryotic cell lines

Policy information about cell lines and Sex and Gender in Research

Cell line source(s)	Sf9 cells for generation of baculovirus and expression of recombinant antibody fragment are from Thermo Fisher (12659017, lot 421973).
Authentication	The cells were not authenticated experimentally for these studies.
Mycoplasma contamination	The cells were tested negative for mycoplasma contamination using the CELLshipper Mycoplasma Detection Kit M-100 from Bionique.
Commonly misidentified lines<br>(See ICLAC register)	No commonly misidentified lines

Animals and other research organisms

Policy information about studies involving animals; ARRIVE guidelines recommended for reporting animal research, and Sex and Gender in Research

Laboratory animals	One-month-old C57BL/6 mice (both male and female) were order from Charles River Laboratories. The housing conditions were set as: temperature 68–72 F, humidity 40–60%, dark/light cycle 12:12 hours. No experimental manipulations were performed on these animals.
Wild animals	This study did not involve wild animals.
Reporting on sex	Mice of equally mixed genders were used for the isolation of native GABAA receptors.
Field-collected samples	This study did not use any field-collected samples.
Ethics oversight	All mice were euthanized under the OHSU Institutional Animal Care and Use Committe (IACUC) protocol — TR03_IP00000905, consistent with the recommendations of the panel on euthanasia of the American Veterinary Medical Association (AVMA) carried out only by members of Dr. Gouaux's lab approved on the IACUC protocol.

Note that full information on the approval of the study protocol must also be provided in the manuscript.
